# Supplementary material for: Mixotrophy emerges as an optimal strategy in mature waters of the Amazon River plume
Source: Commun Biol. 2026 Mar 25;9:434. doi: 10.1038/s42003-026-09893-4 (PMC13022006; doi:10.1038/s42003-026-09893-4)
Supplement: Supplementary file 1 — Supplementary Information [file 42003_2026_9893_MOESM1_ESM.pdf]

Supplementary Information

Mixotrophy emerges as an optimal strategy in mature waters of the Amazon River plume

Ana Fernández-Carrera[1, 2*], Noémie Choisnard[1], Dirk Wodarg[1], Iris Liskow[1], Ajit Subramaniam[3], Joseph P. Montoya[4], Maren Voss[1], Natalie Loick-Wilde[1**]

Affiliations:

[1]Department of Biological Oceanography. Leibniz Institute for Baltic Sea Research Warnemuende, 18119 - Rostock, Germany.

[2]Present address: Institute of Oceanography and Global Change. Universidad de Las Palmas de Gran Canaria, 35214-Taliarte, Spain.

[3]Lamont-Doherty Earth Observatory, Columbia University, 10964 - Palisades NY, USA.

[4]School of Biological Sciences, Georgia Institute of Technology, 30332 - Atlanta GA, USA.

Corresponding authors: *ana.carrera@ulpgc.es, **natalie.loick-wilde@io-warnemuende.de

Term	Definition
Autotroph	An organism capable of generating its own food from dissolved inorganic nutrients and a trophic position of 1.0 for both TP_{Glu} and TP_{Ala}.
Microalgae	A community of photosynthesizing unicellular plankton organisms that are pigmented and include phytoplankton and mixoplankton.
Mixoplankton	A unicellular, eukaryotic, planktonic organism (i.e., a protist) that combines osmo-, phago-, and photoautotrophy. This includes all protists except diatoms.
Mixotroph	An organism that grows by combining photoautotrophy, osmotrophy, and/or phagotrophy and a trophic position of 1.5 TP_{Glu} and/or TP_{Ala}.
Osmotrophy	A nutritional mode by which organisms absorb dissolved organic compounds from their environment, reflecting in a trophic position of 2.0 for TP_{Glu} and of 1.0 for TP_{Ala}, respectively, when it is their sole nutritional mode.
Phagotrophy	A nutritional mode by which organisms obtain nutrients by grazing and ingesting other organisms, reflecting in a trophic position of 2.0 for TP_{Ala} and of 1.0 for TP_{Glu}, respectively, when it is their sole nutritional mode.
Photoautotrophy	A nutritional mode by which organisms use light energy to create their own food from inorganic sources, reflecting in a TP of 1.0 for both TP_{Glu} and TP_{Ala}.
Phytoplankton	A unicellular prokaryotic or eukaryotic primary producer combining only osmo-photoautotrophy, namely cyanobacteria and diatoms, which are unable to phagocytose other organisms.
Trophic Position	The numerical position an organism occupies in a food web. In our study, trophic position (TP) is calculated based on nitrogen isotopes in the trophic amino acids glutamic acid (Glu) or alanine (Ala) versus the source amino acid phenylalanine (Phe), e.g., TP_{Glu} or TP_{Ala}.

Supplementary Table 1. *Glossary of terms used in this manuscript in alphabetical order.*

Supplementary Note 1: Apparent age of the Amazon River plume

During our cruise in 2018 and cruise EN640 in 2019, included in the habitat delineation by Pham et al. (2024)[1], radium isotopes were analyzed underway and in discrete samples taken from the CTD[2,3]. Radium, which desorbs from riverine particles as the salinity increases, has four isotopes with contrasting half-lifes (^{224}Ra is 3.66 d; ^{223}Ra is 11. 4 d; ^{228}Ra is 5.75 yr; ^{226}Ra is 1600 yr), hence the ratios of different isotopes provide chronometers for estimating water residence time[4]. We calculated the apparent age of the plume waters using the activity ratio of ^{224}Ra/^{226}Ra according to the equation proposed by Moore (2000)[5]:

$$t = \frac{LN\left(\frac{[^{224}Ra\ /\ ^{226}Ra]i}{[^{224}Ra\ /\ ^{226}Ra]obs}\right)}{\lambda_{224} - \lambda_{226}} \times 1$$

Where $[^{224}Ra/^{226}Ra]_i$ corresponds to the initial activity ratio at the source of freshwater (i.e., time zero), $[^{224}Ra/^{226}Ra]_{obs}$ corresponds to the observed activity ratio at each sampling point, and λ is the decay constant of each isotope calculated as LN(2) divided by the half-life of the isotope in days. We used an initial ratio of 7, which was estimated by averaging the activity ratio of measured ^{224}Ra and ^{226}Ra in surface waters with salinities between 5-9 on the Amazon shelf in May1990 by Moore et al. (1995)[6]. Because this age is related to an arbitrarily defined time zero on the shelf close to the river mouth, age is apparent and not absolute. The age of the different surface samples is shown in Supplementary Figure 1A, B and is provided in Supplementary Data 3.

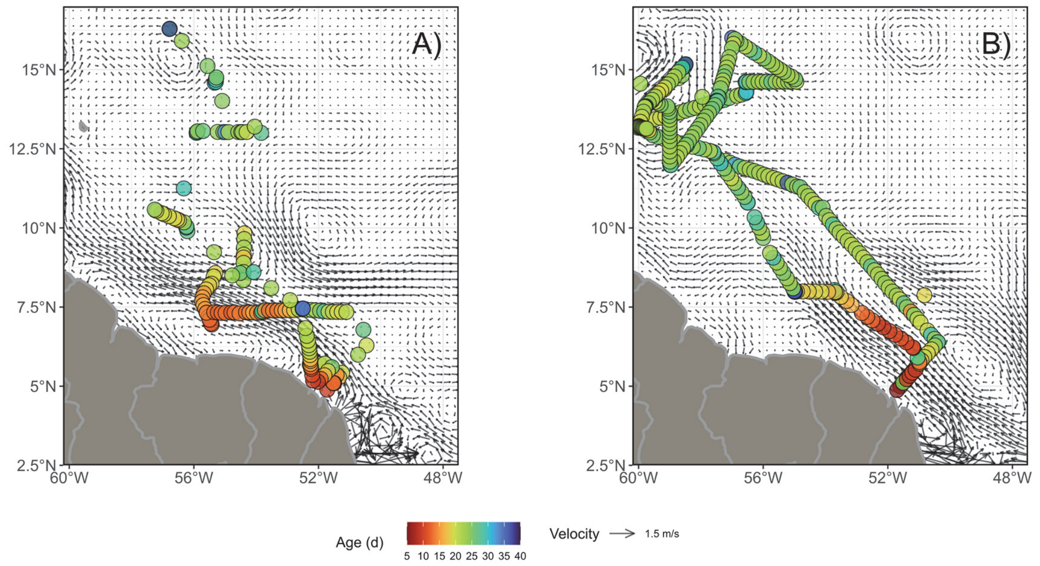

Supplementary Figure 1. Apparent age of surface waters during 2018 (EN614) and 2019 (EN640) cruises along the Amazon River plume. *Color circles represent the apparent age of the plume estimated by radium isotopes measured underway and from CTD samples at surface during our cruise in 2018[2]* ***(A)*** *and in 2019[3]* ***(B)*** *cruise include in Pham et al. (2024)[1] for the habitat delineation along the Amazon River plume. The activity ratio on the Amazon shelf, representing time zero, was estimated using the average of the surface samples at salinities between 5 and 9 in Moore et al. (1995)[6]. The arrows represent the median of the geostrophic velocity provided by the Copernicus Global Ocean Gridded L4 Sea Surface Heights for the duration of each cruise[7]. Data are provided in Supplementary Data 3 for a total of n = 133 and n = 285 individual samples in 2018 and 2019, respectively.*

Supplementary Note 2: Bulk carbon isotopes reveal microalgae as major sources of seston

Seston may contain a mixture of phytoplankton, mixoplankton, microzooplankton, bacteria and detritus, whose contribution can be disentangled using carbon isotopes. The bulk C:N ratio, $\delta^{13}C$, and the relation between particulate carbon (bulk PC) and chlorophyll *a* (Chla) are commonly used to confirm the dominance of microalgae (for the scope of this study, pigmented unicellular plankton

belonging to phyto- and mixoplankton) in the particle signature. Bulk PC showed a linear relationship with Chla concentration measured at the surface (Supplementary Fig. 2A), this significant linear relationship between the log-log of the two variables (two-sided linear regression: bulk PC = 1.0 + 0.4 Chla, n = 27, R^2adj = 0.35, $p = 5.12 \times 10^{-4}$) suggests the dominance of microalgae biomass in the signature of seston (Supplementary Fig. 2A). It is to be noted that all mixotrophic samples showed this relationship between bulk PC and Chla, indicating that the higher trophic position of these samples is unlikely to be simply the result of a mixture of microalgae and herbivorous microzooplankton. Excluding the three autotrophic samples with bulk PC values higher or lower than those with similar Chla content (stations 12.01 and 17.01 in 2018 and 12.02 in 2021), the linear relationship was still significant with a more than twofold increase in R^2adj (two-sided linear regression: bulk PC = 1.1 + 0.5 Chla, n = 24, R^2adj = 0.76, $p = 7.29 \times 10^{-9}$). In addition, the bulk C:N ratio of seston ranged from 6.2 to 16.6, and in the majority of our samples, it is consistent with the ranges described for marine or freshwater algae and organic particulate matter[8] with little or no imprint of bacteria or higher plant matter delivered by the river, including the three samples with anomalous bulk PC-Chla ratios (Supplementary Fig. 2B). These C:N values above 6 also suggest no contribution from microzooplankton in our samples, which typically have C:N ratios below 6.6 [9], and further support the fact that mixotrophic samples are not the result of mixing of different trophic levels in seston, but of the combined autotrophic and heterotrophic metabolism of mixotrophs.

The bulk $\delta^{13}C$ of seston ranged between –31 and –27 ‰ in the RI habitat and between –26 and −17 ‰ in the other habitats (Supplementary Fig. 2B). Values in RI are consistent with previous studies reporting $\delta^{13}C$ values of terrestrial material between −34 and −27 ‰[10–12] in the region, implying that seston in the brackish waters of the river mouth are mostly of fluvial origin with little input from the adjacent marine materials. This fluvial signal observed in brackish waters (salinity < 6) disappears when salinity increases above 15, reflecting a shift to marine particles dominance in subsequent habitats. The depletion of $\delta^{13}C$ depletion in the smaller size fraction relative to the large of 2021 reflects the microalgae group compositions, with picoseston generally more depleted (−26 to −23‰) than nano- and microseston (−24 to −17 ‰) [13]. This difference in the signatures of different microalgae could also explain the higher bulk $\delta^{13}C$ measured at stations 6.04, 8.02 and 22.04 in 2018, and 24.01 in 2021 (above −19 ‰), where we found a dominance of diatoms and some contribution of *Trichodesmium* (Fig. 3C), a species known to have a bulk $\delta^{13}C$ as high as −12.9 ‰ [14]. In 2018, the total bulk samples collected presented a range of variation similar to that of the large size fraction collected in 2021, suggesting that the signature of the community was driven by larger plankton rather than picoplankton. Overall, these data suggest that our samples are also consistent with the bulk $\delta^{13}C$ organic material derived from primary producers (Supplementary Fig. 2B).

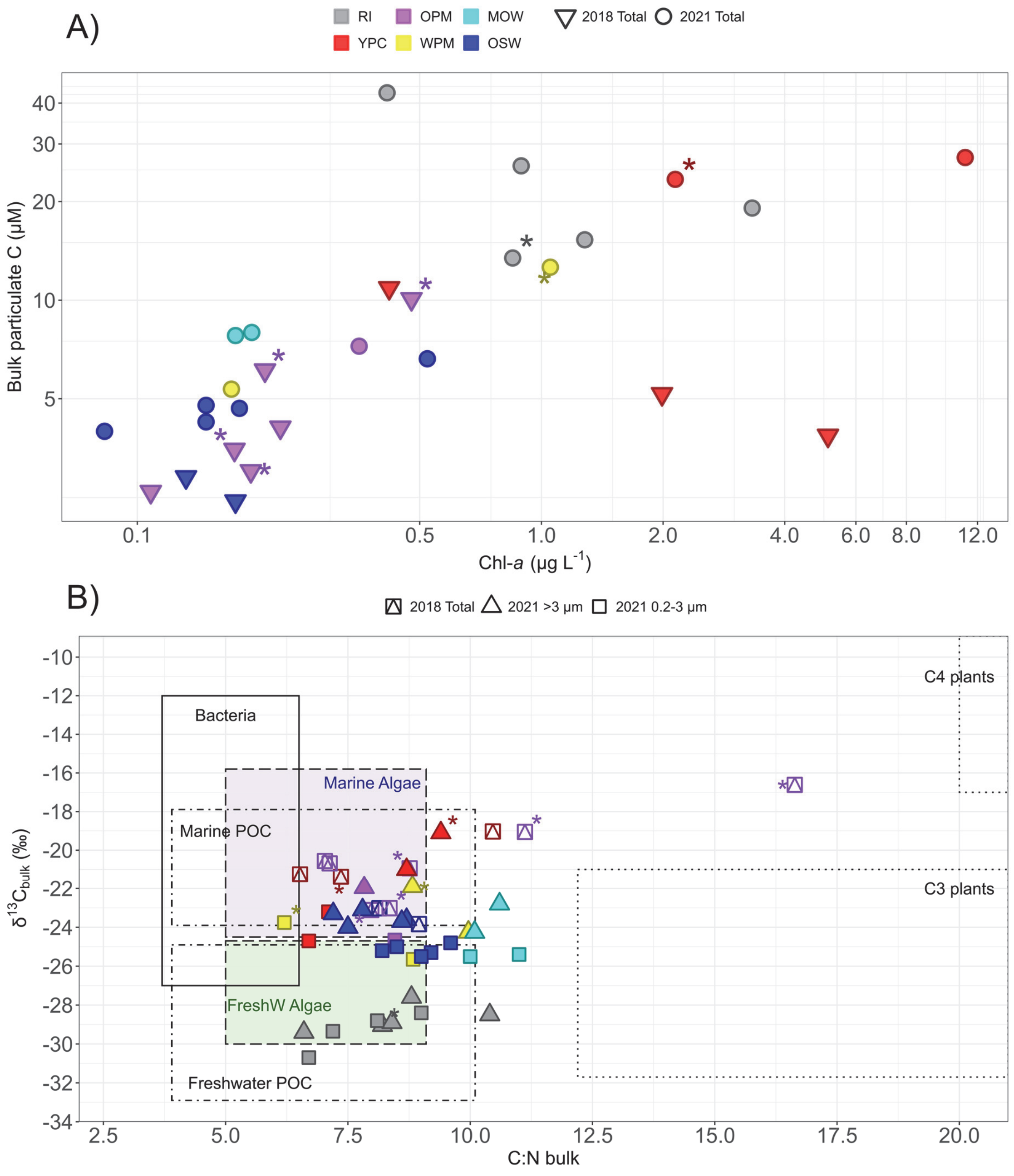

Supplementary Figure 2. Carbon sources driving the signature of seston during 2018 and 2021 cruises along the Amazon River plume*. *A) *Log-log plot of total particulate carbon to chlorophyll* a *with natural values in the scales.* ***B)*** *Bulk carbon isotopes expressed in delta notation ($\delta^{13}C$ ‰ relative to VPDB) vs the C:N ratio, the boxes represent the ranges define in Lamb et al. (2006)[8] for various sources of carbon based on a literature revision. Panel A shows n = 29 data points of total particulate carbon resulting from the combination of 12 individual total samples in 2018 and the sum of the two size fractions collected in 2021. Panel B shows n = 45 individual samples collected at surface. Colors represent the different habitats defined by Pham et al. (2024)[1] ordered by apparent age: Riverine Input (RI, gray), Young Plume Core (YPC, red), Outer Plume Margin (OPM, purple), Western Plume Margin (WPM, yellow), modified Oceanic Water (MOW, cyan) and Oceanic Water (OSW, blue). Mixotrophs (TP_{Glu} 1.5) are marked with an asterisk using the same colors as the symbols.*

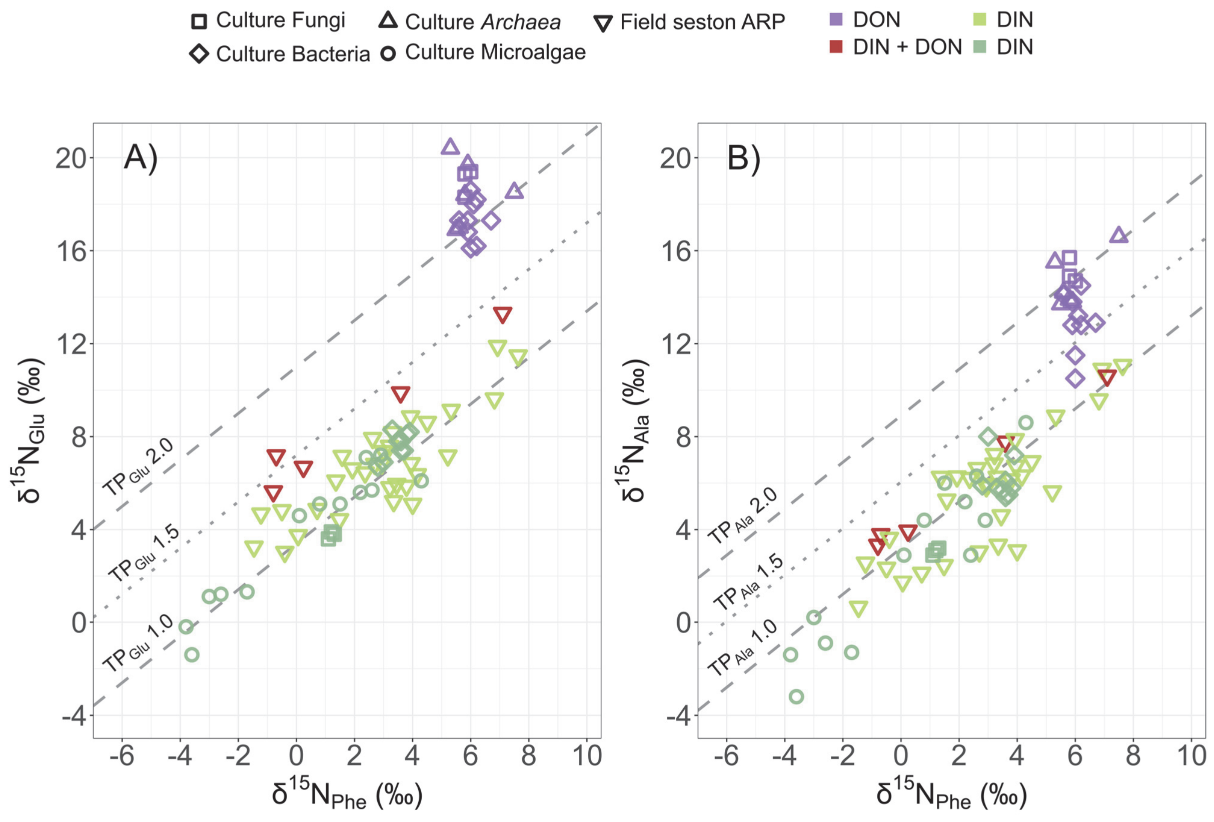

Supplementary Figure 3. Literature end members and seston samples along the Amazon River plume showing autotrophy, osmotrophy and osmo-photoautotrophy. A) *$\delta^{15}N_{Phe}$ (phenylalanine) vs $\delta^{15}N_{Glu}$ (glutamic acid + glutamate) of the literature end members and seston samples collected along the Amazon River plume.* ***B)*** *$\delta^{15}N_{Phe}$ (phenylalanine) vs $\delta^{15}N_{Ala}$ (alanine) of the literature end members (n = 43 individual samples) and seston samples collected along the Amazon River plume (n = 36 individual samples). Symbols represent the organisms: cultured fungi (square), cultured bacteria (diamond), cultured Archaea (triangle), cultured microalgae (circle), and field seston (inverted triangles). Colors represent the nitrogen source: dissolved inorganic nitrogen from culture data (green, DIN), dissolved inorganic nitrogen from our seston data (dark green), dissolved organic nitrogen from culture data (purple), DIN + DON in our seston (dark red). The dashed and dotted gray lines represent the trophoclines like in Figure 1A, B.*

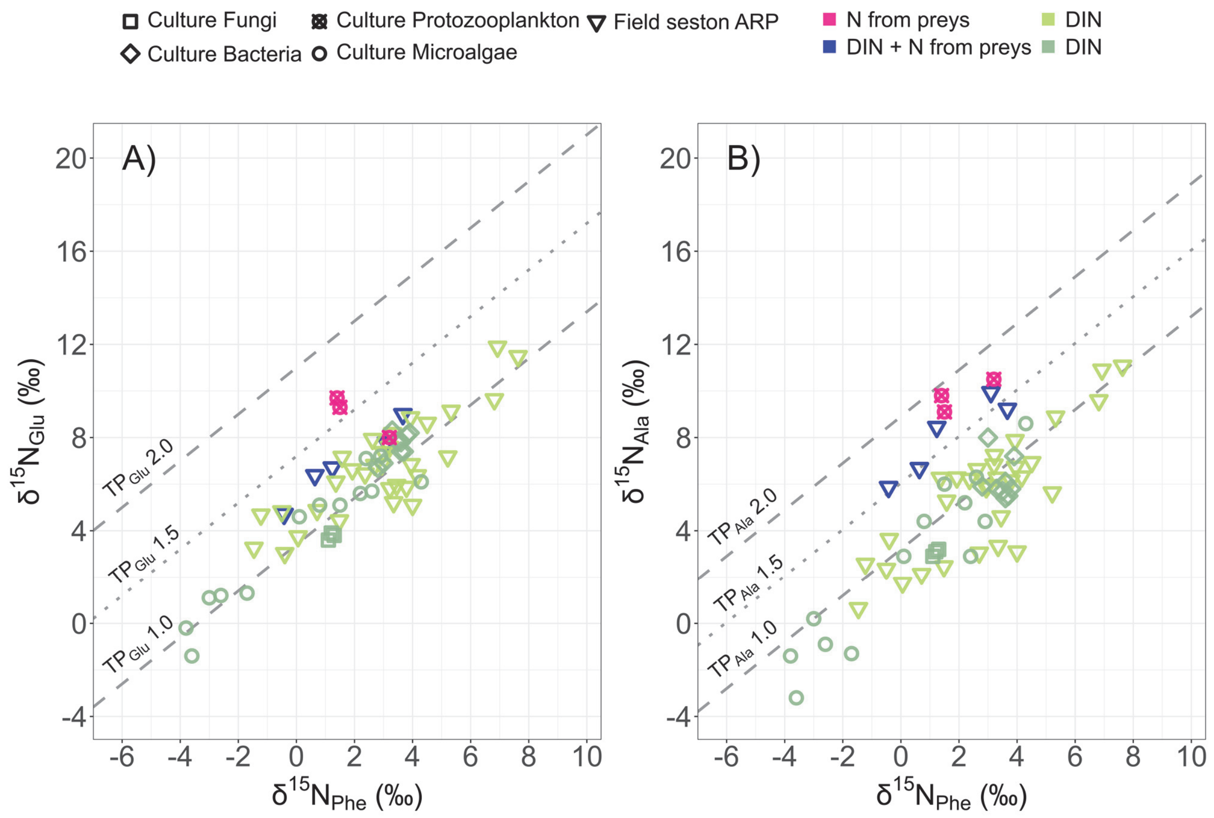

Supplementary Figure 4*. Literature end members and seston samples along the Amazon River plume showing autotrophy, phagotrophy, and phago-photoautotrophy. A)** *$\delta^{15}N_{Phe}$ (phenylalanine) vs $\delta^{15}N_{Glu}$ (glutamic acid + glutamate) of the literature end members and seston samples collected along the Amazon River plume.* ***B) *$\delta^{15}N_{Phe}$ (phenylalanine) vs $\delta^{15}N_{Ala}$ (alanine) of the literature end members (n = 28 individual samples) and seston samples collected along the Amazon River plume (n = 36 individual samples). Symbols represent the organisms: cultured fungi (square), cultured bacteria (diamond), cultured protozooplankton (crossed circle), cultured microalgae (circle), and field seston (inverted triangles). Colors represent the nitrogen source: dissolved inorganic nitrogen in culture data (green), dissolved inorganic nitrogen in our seston data (dark green), DIN + N from phagocytosis (preys) in our seston (blue), N from phagocytosis in culture protozooplankton (pink). The dashed and dotted gray lines represent the trophoclines like in Figure 1A, B.*

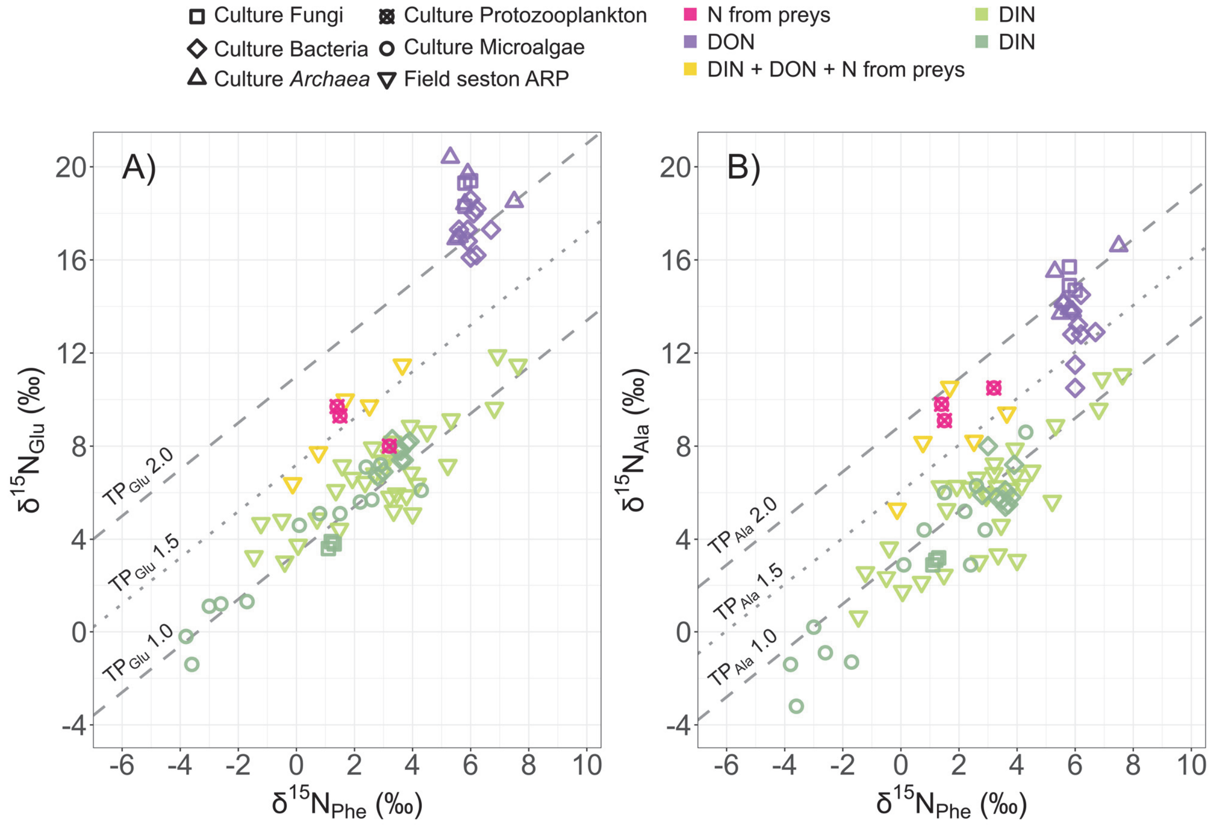

Supplementary Figure 5**. *****Literature end members and seston samples along the Amazon River plume showing autotrophy, osmotrophy, phagotrophy and osmo-phago-photoautotrophy*****. *****A)*** *$\delta^{15}N_{Phe}$ (phenylalanine) vs $\delta^{15}N_{Glu}$ (glutamic acid + glutamate) of the literature end members and seston samples collected along the Amazon River plume.* ***B)*** *$\delta^{15}N_{Phe}$ (phenylalanine) vs $\delta^{15}N_{Ala}$ (alanine) of the literature end members (n = 46 individual samples) and seston samples collected along the Amazon River plume (n = 36 individual samples). Symbols represent the organisms: cultured fungi (square), cultured bacteria (diamond), cultured Archaea (triangle), cultured protozooplankton (crossed circle), cultured microalgae (circle), and field seston (inverted triangles). Colors represent the nitrogen source: dissolved inorganic nitrogen from culture data (green), dissolved inorganic nitrogen from our seston data (dark green), DIN + DON + N from phagocytosis (preys) in our seston (yellow), dissolved organic nitrogen from culture data (purple), and N from phagocytosis in culture protozooplankton (pink). The dashed and dotted gray lines represent the trophoclines like in Figure 1A, B.*

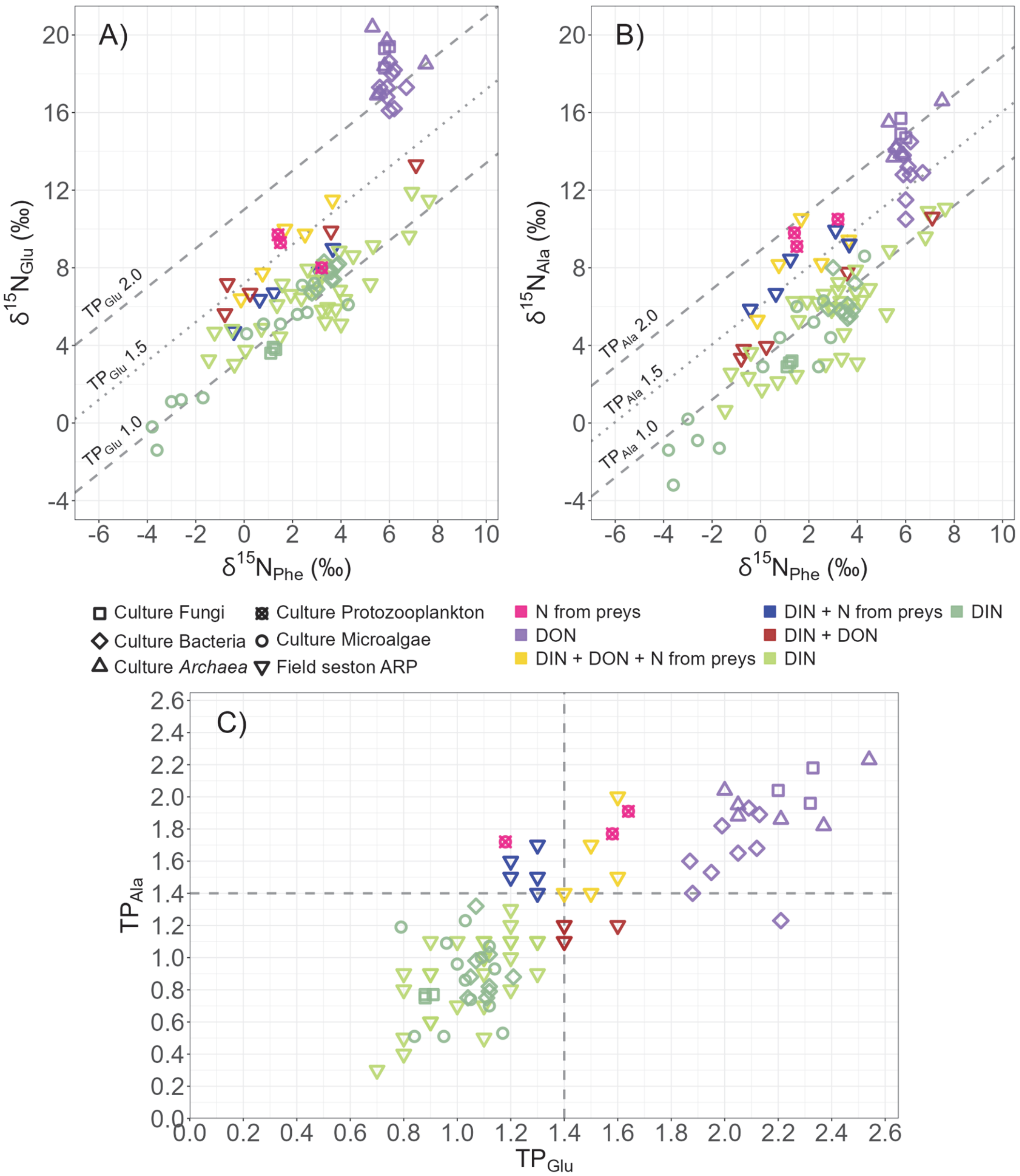

Supplementary Figure 6. Literature end members and seston samples along the Amazon River plume showing all end members. ***A)*** *$\delta^{15}N_{Phe}$ (phenylalanine) vs $\delta^{15}N_{Glu}$ (glutamic acid + glutamate), and* ***B)*** *$\delta^{15}N_{Phe}$ (phenylalanine) vs $\delta^{15}N_{Ala}$ (alanine) of the literature end members (Supporting Data 1, n=46 individual samples) and seston samples collected along the Amazon River plume (Supporting Data 2, n= 46 individual samples).* ***C)*** *Comparison of TP_{Glu} and TP_{Ala} calculated according to the equations proposed by Chikaraishi et al. (2009)*[15]*, the vertical and horizontal dashed lines represent TP_{Glu} 1.4 and TP_{Ala} 1.4, respectively. Symbols represent the organisms: cultured fungi (square), cultured bacteria (diamond), cultured Archaea (triangle), cultured protozooplankton (crossed circle), cultured microalgae (circle), and field seston (inverted triangle). Colors represent the nitrogen source end member: dissolved inorganic nitrogen (green and dark green, DIN), dissolved organic nitrogen (purple, DON), N from the phagocytosis of preys (pink), DIN+ DON (dark red), DIN + phagocytosis (blue), and DIN + DON + phagocytosis (yellow). It should be noted that two seston sample points overlap at TP_{Glu} 1.4 + TP_{Ala} 1.1, and two points overlap at TP_{Glu} 1.4 +TP_{Ala} 1.2 for a total of 5 seston samples on DIN +DON.*

Supplementary Note 3: Stable Isotope Trophic Ecology 101

In this section, we will briefly explain the fundamental concepts of stable isotope ecology and compound-specific isotope analysis of amino acids (CSIA-AA) necessary for understanding the manuscript, which is essential for newcomers. Our primary source on stable isotope ecology is the book Stable Isotope Ecology by Brian Fry [16]. We highly recommend reading more of this book for those interested in learning more about stable isotope ecology. It provides an excellent introduction to the necessary information for entering the field. To the best of our knowledge, there is no book focused on the CSIA-AA. However, a few useful reviews have been published in recent years that should help any reader looking for more information [17–20].

Isotopes are different forms of an element that only differ in the number of neutrons in their nucleus. This difference in neutrons results in subtle differences in chemical properties that make isotopes virtually the same element, as the balance between protons and electrons is what really matters. The actual difference between isotopes lies in their atomic weight, having more neutrons implies a heavier weight. It also implies an unstable nucleus when the number of neutrons is much larger than the number of protons, resulting in a radioactive element. But our focus are stable isotopes. This difference in weight is why isotopes are usually referred to as heavy or light isotope. Heavy isotopes are much less abundant than light isotopes, for example, nitrogen has two stable isotopes: ^{14}N, which accounts for 99.64% of all nitrogen on Earth, and ^{15}N, which accounts for 0.36%[16].

The energetic cost of reactions (i.e., activation energy or the energy hill) will depend directly on the atomic weight. Using a substrate with a heavy isotope is more expensive than using one with a light isotope, this results in a preference for the light isotope whenever a substrate is in excess, known as fractionation. This leads to preferential use of light isotopes in kinetic reactions and concentration of heavy isotopes in strong bonds in exchange reactions. As stated by Fry (2006) [16], these are the two most important rules of isotope ecology. For describing the degree of isotopic fractionation between a substrate and its product, we use the fractionation factor, often reported as an enrichment factor denoted with ε in ‰ (see below for an explanation of these units). To compare two substances, we can use ε to define the difference in the ratio between heavy and light isotopes between them, the so-called apparent isotope fractionation. This apparent fractionation simply reflects how many more or many less ^{15}N isotopes has a substance relative to another.

Fractionation is counteracted by mixing, which basically is the combination of different substances into a homogeneous whole. The final ratio of heavy to light isotopes in the whole results from a weighted average of all the substances. The larger a substance's contribution to the mixture, the greater its influence on the final ratio of heavy to light isotopes.

The δ notation allows us to address all the subtle differences in weight due to isotopes. δ values show the relationship between heavy and light isotopes in reference to a standard, and with numerical values centered at that standard. In other words, positive values represent materials with a greater abundance of heavy isotopes than the standard, while negative values represent materials with a lower abundance. The standard for nitrogen is molecular nitrogen (N_2) in the air, which we now could suspect is very poor in ^{15}N, because the two nitrogen atoms in the molecule are joined by a triple bond. Indeed, the ratio $^{15}N/^{14}N$ ($R_{standard}$) is 0.0036765. The δ values are calculated as:

$$\delta^{15}N = \left(\frac{R_{sample}}{R_{standard}} - 1\right) \times 1000$$

Where R_{sample} represents the ratio of $^{15}N/^{14}N$ in our measured sample. The calculation is similar for all other elements with different stable isotopes (e.g., $\delta^{13}C$, $\delta^{18}O$, etc). The final multiplication by 1000 makes small differences in weight due to neutrons look large. The units of δ are ‰ or permil.

The effect of mixing on the δ of a sample is calculated as a weighted average or mass balance of different end members. Let's illustrate this with a simple theoretical example. Imagine that we have mixed two phytoplankton monocultures with known $\delta^{15}N$, for this example we assume the bulk isotopes of the cultures, but this also applies to a mixture of specific compounds, such as the amino acids explained below. We combined a diatom culture, which was growing on nitrate, with a nitrogen fixing cyanobacterium. The final signature of the mixture will be a weighted mean estimated as:

$$\delta^{15}N_{mixture} = \frac{V_{diatom} \times \delta^{15}N_{diatom} + V_{cyanobacterium} \times \delta^{15}N_{cyanobacterium}}{V_{diatom} + V_{cyanobacterium}}$$

Where V_{diatom} and $V_{cyanobacterium}$ represent the mass or volume of each culture used for the mixture.

Traditionally, trophic hierarchies have been studied using bulk nitrogen isotopes, which are the isotopes of all the nitrogenous compounds in the sample. That is, the trophic position (TP) of an organism is calculated using bulk nitrogen isotopes. In the case of plankton up to 200µm, the focus of our study, bulk isotopes are measured in whole-body samples, which reflect variable timescales in the lifespan of the organisms. For example, microalgae typically have a turnover time of a few days, while metazooplankton may represent a time frame ranging from a few weeks to a few months [21]. This bulk nitrogen isotope approach is thus complicated by potential decoupling of bulk $\delta^{15}N$ signatures given the different N turnover times of autotrophs and consumers [22–24] as well as the origin, movement, and transformation of nitrogen in the upper water column [25–27]. Therefore, it always requires to know the actual baseline (i.e., signature of primary producers) in our systems.

The CSIA-AA overcomes many of the caveats of bulk isotopes by providing information on both TP and nitrogen sources from a single organism/sample. This is because amino acids fall into two functional isotopic groups: trophic amino acids (e.g., glutamic acid, alanine, valine, leucine) that become enriched in ^{15}N with each trophic transfer, and source amino acids (e.g., phenylalanine, lysine) that retain the $\delta^{15}N$ signature of primary producers, that is, the baseline [17]. The simple comparison of trophic and source amino acids allows to define the TP of an organism [28,29]. The canonical pair is usually glutamic acid (Glu) and phenylalanine (Phe), because they have a large and consistent difference across diverse trophic levels [15]. While Glu is enriched in ^{15}N by ~8.0‰ per trophic transfer (the so-called trophic discrimination factor, TDF), the $\delta^{15}N$ of Phe remains nearly unchanged when the amino acid is transferred through the food web (Supplementary Figure 7), always reflecting the isotopic composition of the primary producers (N-source measure, Chikaraishi et al. 2010[30]). This approach largely eliminates potential sources of error in TP estimates associated with temporal and physiological decoupling between a consumer and its diet, and has been refined and confirmed in numerous field- and lab-based trophic studies over the last decade (reviewed by Ohkouchi et al. 2017; Loick-Wilde et al. 2019; Glibert et al. 2019; Weber et al. 2021; and Ohkouchi 2023[17,18,31–33]).

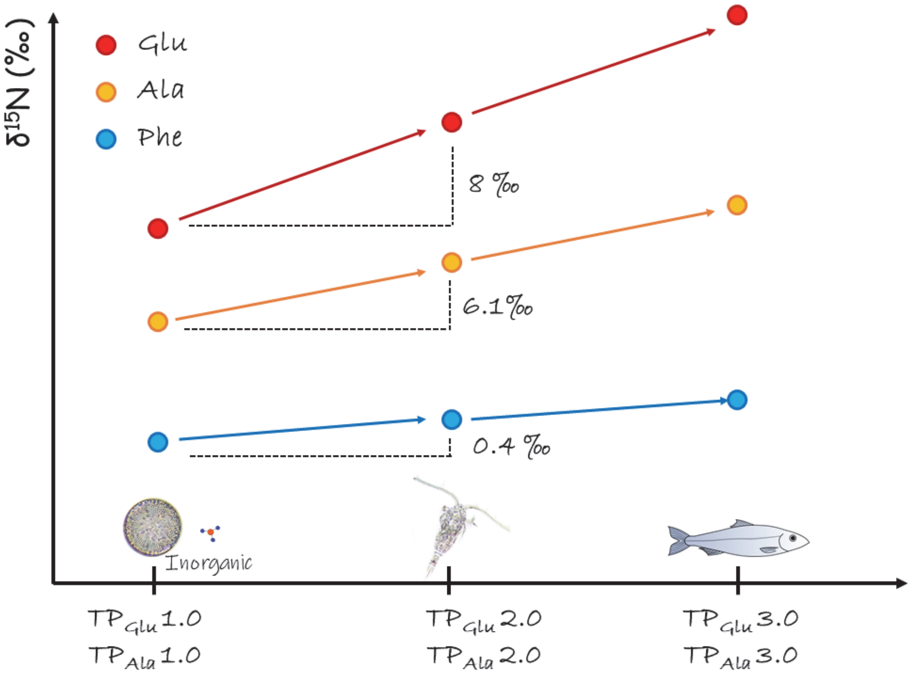

Supplementary Figure 7. *Differential enrichment of glutamic acid or alanine (trophic amino acids) relative to phenylalanine (source amino acid) with trophic transfers in a simple food chain, where an organism illustrates each trophic level: TP1 – diatom, TP2 – copepod, TP3 – generic planktivorous fish. Pictures taken along the Amazon River plume by our PlanktoScope v2.1 during 2021 cruise on board RV Meteor.*

Based on experimental data of different trophic levels, Chikaraishi et al. (2009)[15] proposed the next equation for estimating TP based on the δ^{15}N of Phe and Glu:

$$TP_{Glu} = \frac{\delta^{15}N_{Glu} - \delta^{15}N_{Phe} - 3.4}{TDF_{Glu} - TDF_{Phe}} + 1$$

Where TFD$_{Glu}$ and TDF$_{Phe}$ represent the trophic discrimination factor of Glu and Phe, 8 and 0.4‰, respectively.

For estimating the TP based on δ^{15}N of Phe and Ala, Chikaraishi et al. (2009)[15] proposed:

$$TP_{Ala} = \frac{\delta^{15}N_{Ala} - \delta^{15}N_{Phe} - 3.4}{TDF_{Ala} - TDF_{Phe}} + 1$$

Where TDF$_{Ala}$ is 6.1‰.

It is important to stress that the TP estimated using this approach is based on nitrogen. Therefore, the metabolism of nitrogen related to auto- and heterotrophy is what is relevant in this context, because the fractionation of the amino acids will be a result of the metabolic routes involving movement of amino groups.

Inorganic nitrogen (namely ammonium) is incorporated into organic compounds by autotrophs using two ways: the glutamate pathway or the glutamine pathway [34]. Either by one or the other, the result is that glutamate and glutamine become the nitrogen hub for all the other nitrogenous compounds in organisms. All the nitrogen required for other amino acids, nucleic acids or

nucleotides is hence derived from these two amino acids, and distributed through the different metabolic pathways [34]. Therefore, due to the biological importance of amino acids in all living organisms, their metabolic pathways must be strictly regulated to ensure the basic functioning of these organisms. Within the metabolic routes, only those implying transamination or deamination, that is, cleavage of amino groups, will affect the isotopic signature of individual amino acids. This is because, as we know, a light amino group will be preferentially removed from the substrate towards the product. As a result, the pool of the substrate amino acid is enriched in ^{15}N, while the product amino acid (or nitrogenous compound) is depleted in ^{15}N. These reactions are usually related to the synthesis of amino acids from another, to the supply nitrogen for other compounds, to the production of energy or to excretion routes. For instance, glutamate serves as the direct precursor of aspartate by transamination, that implies the removal of the amino group and transference to another molecule [35]; while the transamination of alanine directly produces pyruvate, which could go into gluconeogenesis, and glutamate, which goes towards biosynthetic pathways or the urea cycle [36]. It is easy to understand that all these reactions mentioned above have an impact on the signature of the trophic amino acids, which will incrementally enrich with every trophic transfer, keeping this way a memory of the previous trophic steps. By contrast, the source amino acids are not typically involved in metabolic routes involving transamination or deamination, and that is why they usually retain the ^{15}N of the baseline barely unchanged.

In this framework, the final ^{15}N signature of the trophic amino acids in microalgae is the result of processes supplying light molecules via autotrophy, and processes enriching the cellular pools via heterotrophy. We could see the whole pool as a black box with inputs/outputs, where the final signature will reflect a weighted mean of all these processes due to mixing, as we explained above. In the case of mixotrophs, combining autotrophy with osmotrophy and/or phagotrophy, the isotopic difference between a given trophic amino acid and a source amino acid will oscillate between that of autotrophs (TP 1.0) towards that of herbivores (TP 2.0). If heterotrophy is more dominant than autotrophy, the apparent isotopic fractionation of the trophic amino acid relative to the source will increase and approach the values in TP 2.0, while when the opposite happens this fractionation will be closer to that of TP 1.0, and when both processes are balanced it should be TP1.5.

Heterotrophic uptake of dissolved organic compounds reflects in glutamic acid nitrogen isotopes. Yamaguchi et al. (2017)[37] compared the effects of fractionation on different amino acids in eukaryotes, bacteria, and *Archaea* that grew on ammonium (chemolithoautotrophically) or casamino acids (heterotrophically). The authors aimed to determine whether heterotrophy affects the nitrogen of amino acids in different prokaryotes and eukaryotes. To determine the most appropriate amino acid combination, the researchers estimated the apparent isotopic fractionation of each amino acid in relation to Glu. They selected Glu as the reference due to its role as a nitrogen hub in the cell. According to the authors, comparing Phe and Glu revealed the ideal pair for tracking the effects of uptake and heterotrophic processing of organic compounds. Microbes growing on ammonium synthesized their own amino acids; therefore, the apparent isotopic fractionation of Phe/Glu was that of TP_{Glu} 1.0. Meanwhile, microbes growing on casamino acids exhibited a difference equal to TP_{Glu} 2.0. Given the central role of Glu in nitrogen metabolism in cells, it is reasonable to expect an effect on nitrogen isotopes due to the uptake and heterotrophic reworking of dissolved, ambient amino acids or organic nitrogen compounds in the field (osmotrophy), similar to that described by Yamaguchi et al. (2017)[37] in laboratory. This effect will reflect in the TP_{Glu},

calculated based on the nitrogen of Glu and Phe, producing a shift in the subsequent trophic levels (Supplementary Figure 8). However, it may not be evident in carbon-based calculations.

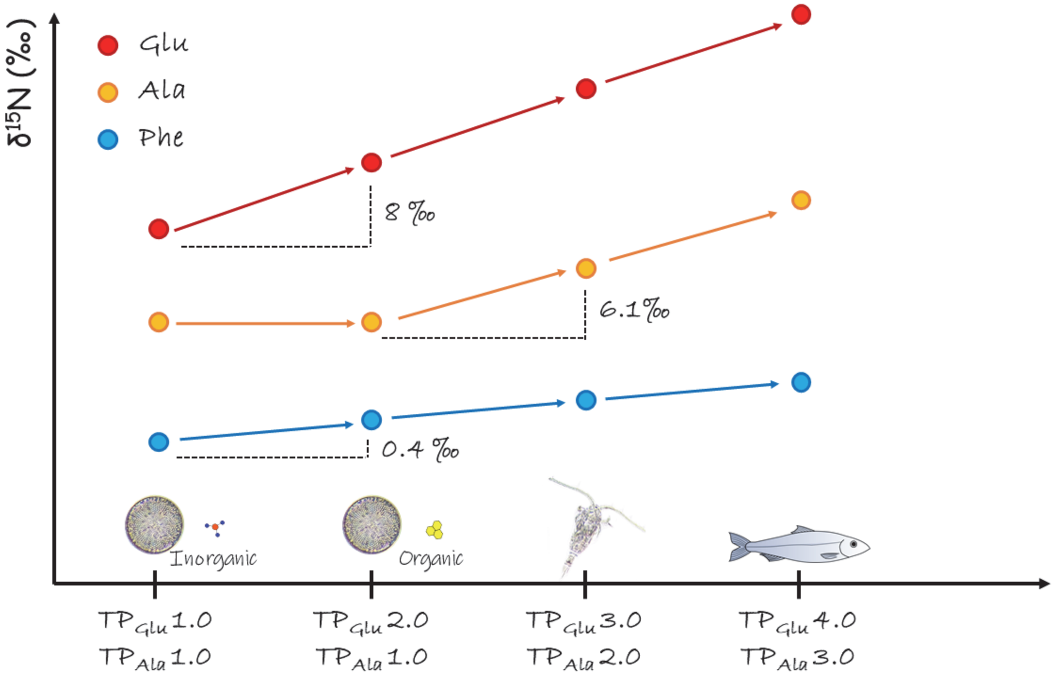

***Supplementary Figure 8**. Differential enrichment of glutamic acid or alanine (trophic amino acids) relative to phenylalanine (source amino acid) with trophic transfers in a simple food chain, where an osmotroph is present. Same organisms as in Supplementary Figure 7. Pictures taken along the Amazon River plume by our PlanktoScope v2.1 during 2021 cruise on board RV Meteor.*

Phagotrophy in heterotrophic protists reflects in alanine nitrogen isotopes. Using laboratory cultures, Gutierrez-Rodriguez et al. (2014)[38] and Décima et al. (2017)[39] found that alanine (Ala) nitrogen isotopes reflects protist grazer activity whereas Glu nitrogen isotopes do not. They emphasized the role of Ala in integrating additional protist grazer trophic levels into the planktonic food web. Though there are a few experimental caveats — such as growing autotrophic prey in continuous light to allow comparison of grazing in darkness versus light or using only eukaryotic prey — the results of these authors were consistent across different protozooplankton taxa (*Oxyrrhis marina*, *Heterocapsa triquetra*, and *Favella* spp.), which grazed on eukaryotic primary producers (*Dunaliella tertiolecta* and *Thalassiosira weissflogii*). Décima et al. (2017)[39] also demonstrated how Ala transfers information about protistan grazing to the next level using a three-step food web (*D. tertiolecta* – *O. marina* – *Calanus pacificus*). Subsequent studies have described the impact of protistan steps on Ala in organisms belonging to different trophic levels [40–42]. The most extensive literature analysis to date was conducted by Viana et al. (2023)[43]. A protistan intermediate step will therefore reflect in a shift of TP_{Ala} in subsequent trophic levels (Supplementary Figure 9).

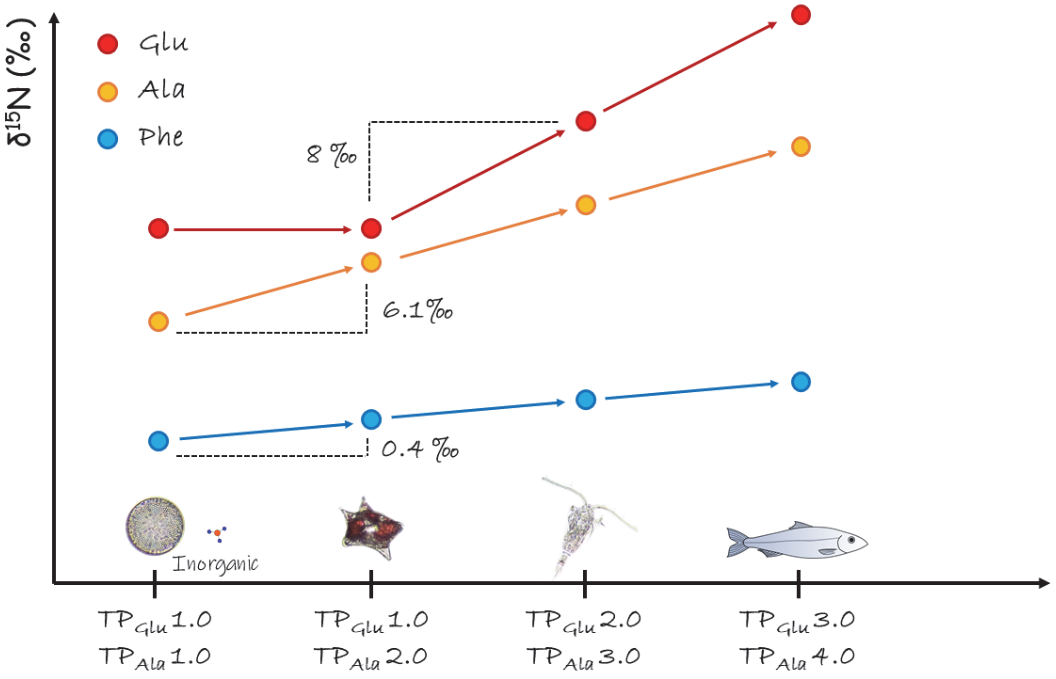

Supplementary Figure 9*. Differential enrichment of glutamic acid or alanine (trophic amino acids) relative to phenylalanine (source amino acid) with trophic transfers in a simple food chain, where a strict phagotrophy (protozooplankton illustrated by a strict heterotrophic dinoflagellate.) is present between the primary producer and the next metazoan consumer. Pictures taken along the Amazon River plume by our PlanktoScope v2.1 during 2021 cruise on board RV Meteor.*

Unfortunately, none of the authors above explored the metabolic basis of the differential nitrogen isotopic enrichment of Ala and Glu in phagotrophs, but it should be possible to hypothesize about the pathways leading to this difference. Alanine is critical in amino acid homeostasis, it is the next most abundant amino acid after aspartate and glutamate, and is central to sugar and amino acid biosynthetic and catabolic pathways [36]. The transamination of Ala results in pyruvate, which goes into fermentation or production of glucose, and glutamate. It is possible that, after digesting prey's peptides, heterotrophic phagotrophs direct a substantial fraction of Ala into this pathway, likely prioritizing energy production with Glu as a by-product. This would enrich the remaining pool of nitrogen isotopes in Ala in the phagotroph, while producing light Glu, which would mask any enrichment due to the transamination or deamination of Glu when supplying nitrogen for other nitrogenous compounds in its metabolic pathways. This is of course a plausible hypothesis based on known metabolic routes in prokaryotes (i.e., *Archaea* and bacteria) and eukaryotes [34,36,44]. However, the specific characterization of the preferential metabolic routes for prey's amino acids in both protozooplankton and mixoplankton is still lacking, and future research is essential to set the foundation of the differential nitrogen isotope enrichment of Ala/Glu found in the aforementioned studies.

Supplementary References

1 Pham AH, Choisnard N, Fernández-Carrera A, Subramaniam A, Strope EK, Carpenter EJ *et al.* Planktonic habitats in the Amazon Plume region of the Western Tropical North Atlantic. Front. Mar. Sci. 2024; **11**. doi:10.3389/fmars.2024.1287497.

2 Peterson, Richard, Montoya, Joseph P., Subramaniam A. Radium isotope measurements from CTD and underway water samples from the R/V Endeavor from 2018-05-06 to 2018-05-29. Biological and Chemical Oceanography Data Management Office (BCO-DMO). (Version 1) Version Date 2019-02-11. 2020. doi:10.26008/1912/bco-dmo.753837.1.

3 Peterson, R. N., Montoya, J., Subramaniam A. Radium isotope (223Ra, 224Ra, and 226Ra) measurements from CTD and underway water samples collected on R/V Endeavor cruise EN640 from June-July 2019. Biological and Chemical Oceanography Data Management Office (BCO-DMO). (Version 1). 2021. doi:10.26008/1912/bco-dmo.846802.1.

4 Léon M, van Beek P, Scholten J, Moore WS, Souhaut M, De Oliveira J *et al.* Use of 223Ra and 224Ra as chronometers to estimate the residence time of Amazon waters on the Brazilian continental shelf. *Limnol Oceanogr* 2022; **67**: 753–767.

5 Moore WS. Ages of continental shelf waters determined from 223Ra and 224Ra. *J Geophys Res Ocean* 2000; **105**: 22117–22122.

6 Moore WS, Astwood H, Lindstrom C. Radium isotopes in coastal waters on the Amazon shelf. *Geochim Cosmochim Acta* 1995; **59**: 4285–4298.

7 E.U. Copernicus Marine Service Information (CMEMS). Copernicus Global Ocean Gridded L4 Sea Surface Heights. 2024. doi:10.48670/moi-00148.

8 Lamb AL, Wilson GP, Leng MJ. A review of coastal palaeoclimate and relative sea-level reconstructions using δ13C and C/N ratios in organic material. *Earth-Science Rev* 2006; **75**: 29–57.

9 Steinberg DK, Saba GK. Chapter 26 - Nitrogen Consumption and Metabolism in Marine Zooplankton. In: Capone DG, Bronk DA, Mulholland MR, Carpenter EJBT-N in the ME (Second E (eds). . Academic Press: San Diego, 2008, pp 1135–1196.

10 Cai D-L, Tan FC, Edmond JM. Sources and transport of particulate organic carbon in the Amazon River and estuary. *Estuar Coast Shelf Sci* 1988; **26**: 1–14.

11 Showers WJ, Angle DG. Stable isotopic characterization of organic carbon accumulation on the Amazon continental shelf. *Cont Shelf Res* 1986; **6**: 227–244.

12 Mortillaro JM, Abril G, Moreira-Turcq P, Sobrinho RL, Perez M, Meziane T. Fatty acid and stable isotope (δ13C, δ15N) signatures of particulate organic matter in the lower Amazon River: Seasonal contrasts and connectivity between floodplain lakes and the mainstem. *Org Geochem* 2011; **42**: 1159–1168.

13 Brandenburg KM, Rost B, Van de Waal DB, Hoins M, Sluijs A. Physiological control on carbon isotope fractionation in marine phytoplankton. *Biogeosciences* 2022; **19**: 3305–3315.

14 Carpenter EJ, Harvey HR, Fry B, Capone DG. Biogeochemical tracers of the marine cyanobacterium Trichodesmium. *Deep Res Part I-Oceanographic Res Pap* 1997; **44**: 27–38.

15 Chikaraishi Y, Ogawa NO, Kashiyama Y, Takano Y, Suga H, Tomitani A *et al.* Determination of aquatic food-web structure based on compound-specific nitrogen isotopic composition of amino acids. *Limnol Oceanogr* 2009; **7**: 740–750.

16 Fry B. *Stable Isotope Ecology*. Springer New York, NY, 2006 doi:https://doi.org/10.1007/0-387-33745-8.

17 Ohkouchi N, Chikaraishi Y, Close HG, Fry B, Larsen T, Madigan DJ *et al.* Advances in the

application of amino acid nitrogen isotopic analysis in ecological and biogeochemical studies. *Org Geochem* 2017; **113**: 150–174.

18 Ohkouchi N. A new era of isotope ecology: Nitrogen isotope ratio of amino acids as an approach for unraveling modern and ancient food web. *Proc Japan Acad Ser B* 2023; **99**: 131–154.

19 Ishikawa NF. Use of compound-specific nitrogen isotope analysis of amino acids in trophic ecology: assumptions, applications, and implications. *Ecol Res* 2018; **33**: 825–837.

20 McMahon KW, Newsome SD. Chapter 7 - Amino Acid Isotope Analysis: A New Frontier in Studies of Animal Migration and Foraging Ecology. In: Hobson KA, Wassenaar LIBT-TAM with SI (Second E (eds). . Academic Press, 2019, pp 173–190.

21 O'Reilly CM, Hecky RE, Cohen AS, Plisnier P-D. Interpreting stable isotopes in food webs: Recognizing the role of time averaging at different trophic levels. *Limnol Oceanogr* 2002; **47**: 306–309.

22 Martínez del Rio C, Wolf N, Carleton SA, Gannes LZ. Isotopic ecology ten years after a call for more laboratory experiments. *Biol Rev* 2009; **84**: 91–111.

23 Montoya JP, Voss M, Capone DG. Spatial variation in N_2-fixation rate and diazotroph activity in the Tropical Atlantic. *Biogeosciences* 2007; **4**: 369–376.

24 Tiselius P, Fransson K. Daily changes in δ15N and δ13C stable isotopes in copepods: equilibrium dynamics and variations of trophic level in the field. *J Plankton Res* 2016; **38**: 751–761.

25 Fry B, Quinones RB. Biomass spectra and stable isotope indicators of trophic level in zooplankton of the northwest Atlantic . *Mar Ecol Prog Ser* 1994; **112**: 201–204.

26 Goering J, Alexander V, Haubenstock N. Seasonal variability of stable carbon and nitrogen isotope ratios of organisms in a North Pacific Bay. *Estuar Coast Shelf Sci* 1990; **30**: 239–260.

27 Layman CA, Araujo MS, Boucek R, Hammerschlag-Peyer CM, Harrison E, Jud ZR *et al.* Applying stable isotopes to examine food-web structure: an overview of analytical tools. *Biol Rev* 2012; **87**: 545–562.

28 McClelland JW, Montoya JP. Trophic relationships and the nitrogen isotopic composition of amino acids in plankton. *Ecology* 2002; **83**: 2173–2180.

29 Mompeán C, Bode A, Latasa M, Fernández-Castro B, Mouriño-Carballido B, Irigoien X. The influence of nitrogen inputs on biomass and trophic structure of ocean plankton: a study using biomass and stable isotope size-spectra. *J Plankton Res* 2016; **38**: 1163–1177.

30 Chikaraishi Y, Ogawa NO, Ohkouchi N. Further evaluation of the trophic level estimation based on nitrogen isotopic composition of amino acids. In: *Earth, Life, and Isotopes*. Kyoto University Press, 2010, pp 37–51.

31 Loick-Wilde N, Fernández-Urruzola I, Eglite E, Liskow I, Nausch M, Schulz-Bull D *et al.* Stratification, nitrogen fixation, and cyanobacterial bloom stage regulate the planktonic food web structure. *Glob Chang Biol* 2019; **25**: 794–810.

32 Glibert PM, Middelburg JJ, McClelland JW, Jake Vander Zanden M. Stable isotope tracers: Enriching our perspectives and questions on sources, fates, rates, and pathways of major elements in aquatic systems. *Limnol Oceanogr* 2019; **64**: 950–981.

33 Weber SC, Loick-Wilde N, Montoya JP, Bach M, Doan-Nhu H, Subramaniam A *et al.* Environmental Regulation of the Nitrogen Supply, Mean Trophic Position, and Trophic Enrichment of Mesozooplankton in the Mekong River Plume and Southern South China Sea.

J Geophys Res Ocean 2021; **126**: e2020JC017110.

34 Bender DA. Nitrogen Metabolism. In: *Amino Acid Metabolism*. 2012, pp 1–65.

35 Bender DA. Amino Acids Synthesized from Glutamate: Glutamine, Proline, Ornithine, Citrulline and Arginine. In: *Amino Acid Metabolism*. 2012, pp 157–223.

36 Peña-Soler E, Fernandez FJ, López-Estepa M, Garces F, Richardson AJ, Quintana JF *et al.* Structural Analysis and Mutant Growth Properties Reveal Distinctive Enzymatic and Cellular Roles for the Three Major L-Alanine Transaminases of Escherichia coli. *PLoS One* 2014; **9**: e102139.

37 Yamaguchi YT, Chikaraishi Y, Takano Y, Ogawa NO, Imachi H, Yokoyama Y *et al.* Fractionation of nitrogen isotopes during amino acid metabolism in heterotrophic and chemolithoautotrophic microbes across Eukarya, Bacteria, and Archaea: Effects of nitrogen sources and metabolic pathways. *Org Geochem* 2017; **111**: 101–112.

38 Gutierrez-Rodriguez A, Decima M, Popp BN, Landry MR. Isotopic invisibility of protozoan trophic steps in marine food webs. *Limnol Oceanogr* 2014; **59**: 1590–1598.

39 Décima M, Landry MR, Bradley CJ, Fogel ML. Alanine δ15N trophic fractionation in heterotrophic protists. *Limnol Oceanogr* 2017; **62**: 2308–2322.

40 Landry MR, Décima MR. Protistan microzooplankton and the trophic position of tuna: quantifying the trophic link between micro- and mesozooplankton in marine foodwebs. *ICES J Mar Sci* 2017; **74**: 1885–1892.

41 Décima M, Landry MR. Resilience of plankton trophic structure to an eddy-stimulated diatom bloom in the North Pacific Subtropical Gyre. *Mar Ecol Prog Ser* 2020; **643**: 33–48.

42 Bode A, Olivar MP, López-Pérez C, Hernández-León S. The microbial contribution to the trophic position of stomiiform fishes. *ICES J Mar Sci* 2021; **78**: 3245–3253.

43 Viana IG, García-Seoane R, Bode A. A missing trophic link: Contribution of the microbial loop to the estimation of the trophic position of pelagic consumers. *Limnol Oceanogr* 2023; **68**: 2587–2602.

44 Nelson D, Cox M. *Lehninger Principles of Biochemistry*. W. H. Freeman, 2021.
