# Supplementary material for: Mixotrophy emerges as an optimal strategy in mature waters of the Amazon River plume
Source: Commun Biol. 2026 Mar 25;9:434. doi: 10.1038/s42003-026-09893-4 (PMC13022006; doi:10.1038/s42003-026-09893-4)
Supplement: Supplementary file 2 — Description of supplementary material [file 42003_2026_9893_MOESM2_ESM.docx]

Description of Additional Supplementary File

File name: Supplementary Data 1
Description: Data 1 contains the literature data for Fig 1 and Supplementary Figs 4-6.

File name: Supplementary Data 2
Description: Data 2 contains the environmental variables used for the machine learning, the numeric TP_Glu_ and TP_Ala_, and the δ15N of the three amino acids used for making Figure 4 and Supplementary Figs 4-6.

File name: Supplementary Data 3
Description: Data 3 contains the apparent age used for Supplementary Figure 1.
