# Supplementary material for: Mixotrophy emerges as an optimal strategy in mature waters of the Amazon River plume
Source: Commun Biol. 2026 Mar 25;9:434. doi: 10.1038/s42003-026-09893-4 (PMC13022006; doi:10.1038/s42003-026-09893-4)
Supplement: Supplementary file 6 — Reporting Summary [file 42003_2026_9893_MOESM6_ESM.pdf]

nature portfolio

Corresponding author(s): Natalie Loick-Wilde and Ana Fernández Carrera

Last updated by author(s): Feb 10, 2026

Reporting Summary

Nature Portfolio wishes to improve the reproducibility of the work that we publish. This form provides structure for consistency and transparency in reporting. For further information on Nature Portfolio policies, see our Editorial Policies and the Editorial Policy Checklist.

Statistics

For all statistical analyses, confirm that the following items are present in the figure legend, table legend, main text, or Methods section.

n/a	Confirmed	
☐	☒	The exact sample size (*n*) for each experimental group/condition, given as a discrete number and unit of measurement
☐	☒	A statement on whether measurements were taken from distinct samples or whether the same sample was measured repeatedly
☐	☒	The statistical test(s) used AND whether they are one- or two-sided<br>*Only common tests should be described solely by name; describe more complex techniques in the Methods section.*
☒	☐	A description of all covariates tested
☒	☐	A description of any assumptions or corrections, such as tests of normality and adjustment for multiple comparisons
☐	☒	A full description of the statistical parameters including central tendency (e.g. means) or other basic estimates (e.g. regression coefficient) AND variation (e.g. standard deviation) or associated estimates of uncertainty (e.g. confidence intervals)
☐	☒	For null hypothesis testing, the test statistic (e.g. *F*, *t*, *r*) with confidence intervals, effect sizes, degrees of freedom and *P* value noted<br>*Give P values as exact values whenever suitable.*
☒	☐	For Bayesian analysis, information on the choice of priors and Markov chain Monte Carlo settings
☐	☒	For hierarchical and complex designs, identification of the appropriate level for tests and full reporting of outcomes
☒	☐	Estimates of effect sizes (e.g. Cohen's *d*, Pearson's *r*), indicating how they were calculated

Our web collection on statistics for biologists contains articles on many of the points above.

Software and code

Policy information about availability of computer code

Data collection	No software was used for data collection.
Data analysis	Statistical analysis and plots were made by open-source software R v4.0.586 in R Studio v2022.02.087. Packages used included: ggplot2 v3.5.1, ggpubr v0.4.0, ncdf4 v1.2490, dplyr v 1.1.491, metR v0.18.292, gganimate v1.0.119, factoextra v1.0.7, FactoMineR v2.4 and C50 v0.1.696.

For manuscripts utilizing custom algorithms or software that are central to the research but not yet described in published literature, software must be made available to editors and reviewers. We strongly encourage code deposition in a community repository (e.g. GitHub). See the Nature Portfolio guidelines for submitting code & software for further information.

Data

Policy information about availability of data

All manuscripts must include a data availability statement. This statement should provide the following information, where applicable:

- Accession codes, unique identifiers, or web links for publicly available datasets
- A description of any restrictions on data availability
- For clinical datasets or third party data, please ensure that the statement adheres to our policy

All data supporting the findings of this study are available within the paper and its Supplementary Information or deposited in open access repositories. Literature data are provided in Supplementary Data 1, along with the original references. Environmental data, trophic positions and δ15N of glutamic acid, alanine and

phenylalanine are provided in Supplementary Data 2. The apparent age of the waters along the Amazon River plume is provided in Supplementary Data3. The Supplementary Movies illustrating the dynamism along the Amazon River plume are available in FigShare with the identifier https://doi.org/10.6084/m9.figshare.31281934. The nitrogen and carbon isotopes of the bulk samples, the nitrogen isotopes and mol% of all amino acids analyzed, the percent contribution of microalgae groups to total chlorophyll a as well as the environmental variables are available in Pangaea98–100. Raw HPLC pigments are available in Subramaniam (2020)101 and Umbricht et al. (2022)102. The PlanktoScope v2.1 images used for validating the classification made by CHEMTAX can be accessed in https://ecotaxa.obs-vlfr.fr/prj/6346.
Fernández-Carrera, Ana; Wodarg, Dirk; Montoya, Joseph P; Loick-Wilde, Natalie (2026): Stable isotopes and mol% of amino acids in seston at surface during ENDEAVOR cruise EN614 [dataset]. PANGAEA, https://doi.pangaea.de/10.1594/PANGAEA.971946
Fernández-Carrera, Ana; Steinkopf, Markus; Liskow, Iris; Wodarg, Dirk; Voss, Maren; Loick-Wilde, Natalie (2026): Stable isotopes and mol% of amino acids in 3-200 µm seston at surface during METEOR cruise M174 [dataset]. PANGAEA, https://doi.pangaea.de/10.1594/PANGAEA.971279
Fernández-Carrera, Ana; Steinkopf, Markus; Liskow, Iris; Wodarg, Dirk; Voss, Maren; Loick-Wilde, Natalie (2026): Stable isotopes and mol% of amino acids in 0.2-3 µm seston at surface during METEOR cruise M174 [dataset]. PANGAEA, https://doi.pangaea.de/10.1594/PANGAEA.971293

Research involving human participants, their data, or biological material

Policy information about studies with human participants or human data. See also policy information about sex, gender (identity/presentation), and sexual orientation and race, ethnicity and racism.

Reporting on sex and gender	This information was not collected because this work does not involve human participants.
Reporting on race, ethnicity, or other socially relevant groupings	N/A
Population characteristics	N/A
Recruitment	N/A
Ethics oversight	N/A

Note that full information on the approval of the study protocol must also be provided in the manuscript.

Field-specific reporting

Please select the one below that is the best fit for your research. If you are not sure, read the appropriate sections before making your selection.

☐ Life sciences ☐ Behavioural & social sciences ☒ Ecological, evolutionary & environmental sciences

For a reference copy of the document with all sections, see nature.com/documents/nr-reporting-summary-flat.pdf

Ecological, evolutionary & environmental sciences study design

All studies must disclose on these points even when the disclosure is negative.

Study description	We conducted two oceanographic research cruises in April/May 2018 and 2021 for studying trophic hierarchies at the base of the planktonic food webs along the Amazon River plume. Our MS is focused on the analysis of suspended particles (seston) as a proxy for autotrophs and mixotrophs.
Research sample	Samples were collected either by Go-Flo or Niskin bottles attached to a CTD-rosette at discrete depths or by an air pump deployed at surface. Samples for particles included in the manuscritp were only taken at the surface within the plume to assess changes in trophic function as different habitats emerge when the plume ages.
Sampling strategy	No sample size calculation was performed. According to the Central Limit Theorem, our initial target was to collect a minimum of 30 samples along the Amazon River plume in order to achieve a normal distribution in our sample size. Both research cruises aimed to cover the largest possible area of the Amazon River plume. Due to diplomatic authorizations in the Exclusive Economic Zone (EEZ), research was conducted using a predefined sampling grid. For areas affected by the plume upstream in international waters, research was conducted based on almost real-time satellite imagery.
Data collection	Each operator recorded metadata por sampling the parameters they were responsible of into hardcopy logsheests and electronic logsheets made in Microsoft Excel following established protocols. Data and metadata were collected and reported following the guidelines of the repositories where they are openly accesible. Literature datasets were collected from published studies either by open repositores or the tables included in the articles cited in the manuscript.Literature datasets were collected from published studies either by open repositores or the tables included in the articles cited in the manuscript.
Timing and spatial scale	Sampling took place during expeditions EN614 on board RV Endeavor and M174 on board RV Meteor. In 2018, the Endeavor departed from Bridgetown (Barbados) on May 8th and arrived in San Juan (Puerto Rico) on June 1st. In this survey, a total of 67 CTD casts were carried out at 19 stations covering different regions and age stages of the ARP. In 2021, the Meteor departed from Las Palmas on April 12th and arrived in Emden (Germany) on May 30th. The survey along the ARP began on April 21st and ended on May 13th, and a total of 114 CTD casts were carried out at 23 stations from the mouth of the Amazon and Pará rivers to the waters east

	Barbados to cover the largest possible geographical extension of the plume. For the scope of this study, we collected suspended particles at surface at 12 stations during 2018 and 17 during 2021, accounting for a total of 46 samples.
Data exclusions	No data were excluded from the analysis.
Reproducibility	Both cruises followed the same established sampling protocols and occurred during a similar period of Amazon River outflow.
Randomization	This is a field study, randomization is not relevant.
Blinding	Blinding does not apply to our study.

Did the study involve field work? ☒ Yes ☐ No

Field work, collection and transport

Field conditions	Environmental variables for each station (temperature, salinity, inorganic nutrients...) are provided in Supporting Data 2 and the open datasets available in Pangaea.
Location	29 stations along the Amazon River plume from 0.6S 48.3W to 16.3N 56.8W. Exact locations and sampling depths can be found in Supporting Data 2.
Access & import/export	Diplomatic applications for working in the EEZ of Brazil, French Guiana and Barbados were granted by the corresponding national governments.
Disturbance	No disturbance was caused during our study.

Reporting for specific materials, systems and methods

We require information from authors about some types of materials, experimental systems and methods used in many studies. Here, indicate whether each material, system or method listed is relevant to your study. If you are not sure if a list item applies to your research, read the appropriate section before selecting a response.

Materials & experimental systems

n/a	Involved in the study	
☒	☐	Antibodies
☒	☐	Eukaryotic cell lines
☒	☐	Palaeontology and archaeology
☒	☐	Animals and other organisms
☒	☐	Clinical data
☒	☐	Dual use research of concern
☒	☐	Plants

Methods

n/a	Involved in the study	
☒	☐	ChIP-seq
☒	☐	Flow cytometry
☒	☐	MRI-based neuroimaging

Plants

Seed stocks	N/A
Novel plant genotypes	N/A
Authentication	N/A
